# New Concept of Intraoperative Fluorescence Diagnostics for Photodynamic Dosimetry of Tumors Using Chlorin-Based Photosensitizers

**DOI:** 10.3390/bios16090526

**Published:** 2026-09-20

**Authors:** Kanamat Efendiev, Arkadii Moskalev, Polina Alekseeva, Alexander Borodkin, Alexey Skobeltsin, Vladimir Makarov, Nina Kalyagina, Kirill Linkov, Aida Gilyadova, Olga Shpileva, Dinara Ilyasova, Tatiana Pisareva, Polina Kozlova, Aidai Rakhmanova, Artem Shiryaev, Victor Loschenov

**Affiliations:** 1 Department of Medical Photonics, Prokhorov General Physics Institute of the Russian Academy of Sciences, 119991 Moscow, Russiaalekseevapolina@nsc.gpi.ru (P.A.); vi.makarov@physics.msu.ru (V.M.); loschenov@nsc.gpi.ru (V.L.); 2 Department of Laser Micro-, Nano-, and Biotechnology, Institute of Engineering Physics for Biomedicine, National Research Nuclear University “MEPhI”, 115409 Moscow, Russia; 3 Institute of Mathematics and Natural Sciences, Kabardino-Balkarian State University, 360004 Nalchik, Russia; 4 Laboratory of Neurobiology and Tissue Engineering, Brain Science Institute, Research Center of Neurology, 125367 Moscow, Russia; 5 Department of Oncology, Radiotherapy and Reconstructive Surgery, Levshin Institute of Cluster Oncology, Sechenov First Moscow State Medical University, 119435 Moscow, Russia

**Keywords:** fluorescence diagnostics, optical clinical diagnostics, photodynamic therapy, endoscopic video system, chlorin-based photosensitizer, chlorin e6, basal cell carcinoma, ovarian cancer, peritoneal carcinomatosis, vulvar intraepithelial neoplasia

## Abstract

Photodynamic therapy (PDT) with chlorin-based photosensitizers requires real-time monitoring of photosensitizer distribution and photobleaching to guide treatment, yet existing endoscopic fluorescence systems rely on short-wavelength (375–450 nm) excitation that is spectrally incompatible with 660 nm chlorin e6 (Ce6)-PDT, forcing interruption of therapy for diagnostic imaging. We developed VENERA-red-660, a dual-channel endoscopic system using a single 660 nm laser for simultaneous white-light and Ce6 fluorescence imaging (>670 nm detection) without interrupting irradiation. System sensitivity was validated on optical phantoms (Ce6 0.1–5.0 mg/L), and phototheranostics was performed in patients with basal cell carcinoma (*n* = 5), vulvar intraepithelial neoplasia grade III (*n* = 3), and peritoneal carcinomatosis (*n* = 10) using intravenous chlorin-based photosensitizer. Phantom testing showed a linear fluorescence response (R^2^ = 0.998) down to the lowest tested concentration of 0.1 mg/L. Pre-PDT fluorescence contrast indices confirmed photosensitizer selectivity in all three tumor types, decreasing by 57%, 66%, and 60% after treatment, consistent with photobleaching; continuous intra-PDT monitoring confirmed progressive photobleaching kinetics, and confocal microscopy verified cellular-level Ce6 selectivity. Increased backscattered laser signal after PDT was consistent with treatment-induced changes in tissue optical properties. These pilot findings demonstrate that single-source phototheranostic monitoring is feasible across external and laparoscopic access routes, supporting further evaluation of VENERA-red-660 as a tool for intraoperative PDT dosimetry.

## 1. Introduction

Photodynamic therapy (PDT) is a promising treatment modality for malignant neoplasms, based on the selective accumulation of a photosensitizer (PS) in tumor tissue, followed by its activation with light of a specific wavelength. This triggers a cascade of photochemical reactions resulting in the generation of reactive oxygen species (ROS) that mediate the photodynamic effect. PDT exerts its therapeutic action through several complementary mechanisms: direct cytotoxic damage to tumor cells via the induction of apoptosis, necrosis, and/or autophagy; destruction of the tumor vasculature with subsequent disruption of the tumor blood supply; and activation of an antitumor immune response [1,2,3,4]. The combination of these mechanisms ensures high therapeutic selectivity, minimal systemic toxicity, and the feasibility of repeated applications, making PDT a highly relevant and promising tool in the treatment of tumors at various anatomical sites, including basal cell carcinoma (BCC), peritoneal carcinomatosis (PC), and vulvar intraepithelial neoplasia (VIN) [5,6,7].

A critical prerequisite for effective PDT is personalized monitoring of PS distribution in tumor tissue and accurate assessment of the light dose [8]. One method of indirect PDT dosimetry is the evaluation of PS photobleaching based on changes in its fluorescence intensity. Fluorescence navigation enables real-time visualization of PS accumulation zones, thereby enhancing the precision of intraoperative planning and PDT monitoring [9].

**Figure 1:**
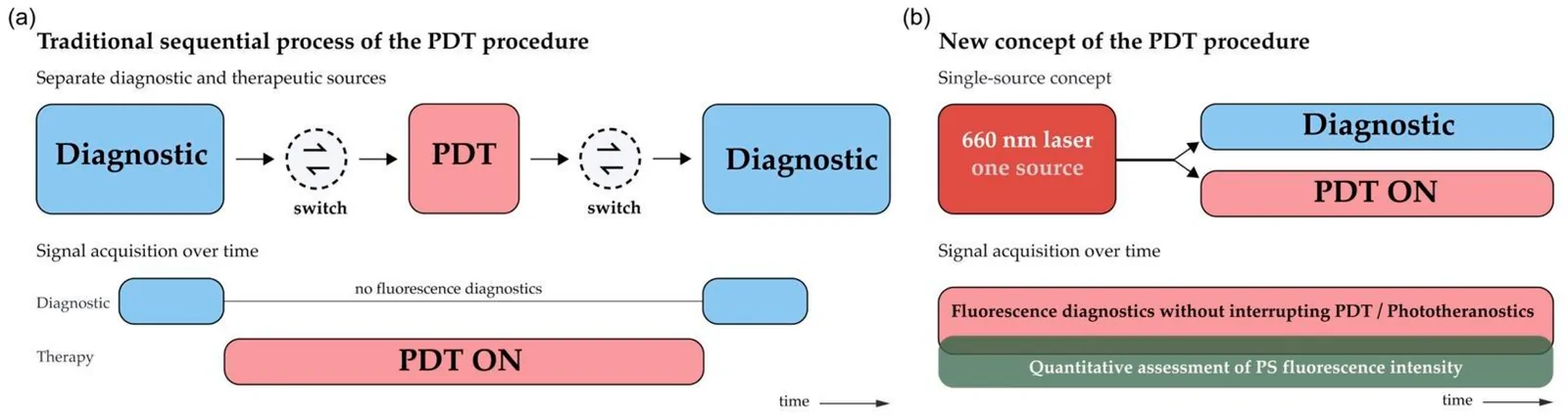
Single-source concept of simultaneous phototheranostics during Ce6-mediated PDT using VENERA-red-660. (**a**) Conventional sequential workflow: diagnostic and therapeutic sources are switched, interrupting fluorescence diagnostics for the entire duration of PDT irradiation. (**b**) The proposed single-source concept: the same 660 nm laser beam simultaneously drives PDT and Ce6 fluorescence excitation, enabling continuous, quantitative fluorescence diagnostics throughout PDT without interrupting irradiation or switching optical sources.

Currently, chlorin-based photosensitizers (PSs) with chlorin e6 (Ce6) as the active ingredient are among the most widely used agents in clinical practice for the PDT of various tumors. Video-fluorescence imaging [10,11,12], fluorescence spectroscopy [13], a combination thereof [14], and diffuse reflectance spectroscopy [15] are the methods most frequently used for Ce6-PDT dosimetry. Ce6 is characterized by an intense absorption peak in the 400–405 nm range (Soret band) and a less pronounced Q–band in the 650–670 nm range [16]. Currently available endoscopic fluorescence imaging systems provide PS fluorescence excitation in the Soret band within the UV/blue spectral range (375–450 nm) or around 635 nm (summarized in Discussion section). We have previously reported a related dual-channel video-fluorescence system operating with a 635 nm diagnostic laser and a spectrally separate 663 nm therapeutic laser [10,17]; because fluorescence excitation and PDT irradiation relied on two distinct sources in that configuration, diagnostic and therapeutic modes still had to be switched, and the system was not suited for real-time monitoring of the Ce6-PDT process itself (Figure 1a).

In contrast, the key distinguishing feature of the system reported here is that a single 660 nm laser simultaneously serves as both the therapeutic and the fluorescence-excitation source, enabling continuous dual-channel (white-light and fluorescence) acquisition throughout PDT without switching between diagnostic and therapeutic modes—a capability not offered by other endoscopic fluorescence platforms (Figure 1b).

The presence of an absorption band in the longer-wavelength region (around 660 nm) and fluorescence in the far-red range (above 665 nm) in chlorin-based PSs can be utilized to detect tumors in deeper tissue layers [18]. Previously, we demonstrated the feasibility of fluorescence spectroscopy with Ce6 excitation at 660 nm and fluorescence detection in the near-infrared (NIR) range of 725–780 nm [13]. The wavelength range of 650–950 nm corresponds to the first NIR optical window (NIR-I) [19]. The penetration depth of 400–450 nm radiation is limited to 1–1.5 mm, whereas the penetration depth of 650–700 nm radiation reaches 4.75–5.25 mm [20].

At the same time, a widespread perception in clinical practice is that diagnostic and therapeutic visible-spectrum laser radiation penetrates biological tissue to a depth of no more than 1–2 mm; consequently, fluorescence diagnostics and PDT of deep-seated and large-volume tumors are traditionally considered ineffective [21]. To induce a photodynamic effect in deeper tissue layers, it is essential to deliver a light dose sufficient for generating reactive oxygen species throughout the entire thickness of the tumor lesion [22]. Although this limitation can potentially be overcome by increasing the laser power density, this approach carries a risk of thermal damage to surrounding tissues [23]. Alternative strategies to enhance penetration depth include increasing the laser beam diameter [20,24] or utilizing a longer-wavelength phototherapeutic window of 600–850 nm [19]: an approach implemented in the present study by exciting Ce6 fluorescence at 660 nm. However, it must be taken into account that as laser radiation propagates through tissue, it attenuates with depth due to absorption and scattering; furthermore, owing to the optical heterogeneity of tissue layers, this attenuation is non-linear [24,25], resulting in a lower actual light dose in deep tumor layers compared to superficial zones [26,27]. Nevertheless, even a reduced dose of 3–5 J/cm^2^, which is insufficient for complete tumor tissue destruction, may be adequate to induce immunogenic cell death, repolarize tumor-associated macrophages from the M2 phenotype to the pro-inflammatory M1 phenotype, and consequently activate an antitumor immune response [28,29].

The key conceptual feature of VENERA-red-660 is the dual use of the 660 nm therapeutic radiation: the same laser beam that activates Ce6 and induces the photodynamic effect simultaneously serves as the excitation source for fluorescence monitoring (Figure 1b). Consequently, treatment and diagnostics are performed concurrently, without switching optical sources or interrupting PDT irradiation.

Thus, implementing the concept of fluorescence diagnostics to assess the accumulation and photobleaching processes of chlorin-based PSs with fluorescence excitation at 660 nm may enhance the detection efficacy and photodynamic dosimetry of deep-seated tumor lesions. The system was applied for intraoperative fluorescence diagnostics for photodynamic dosimetry of BCC, VIN grade III (VIN III), and PC, demonstrating for the first time the feasibility of this approach in a clinical setting.

## 2. Materials and Methods

### 2.1. Dual-Channel Video-Fluorescence System

**Figure 2:**
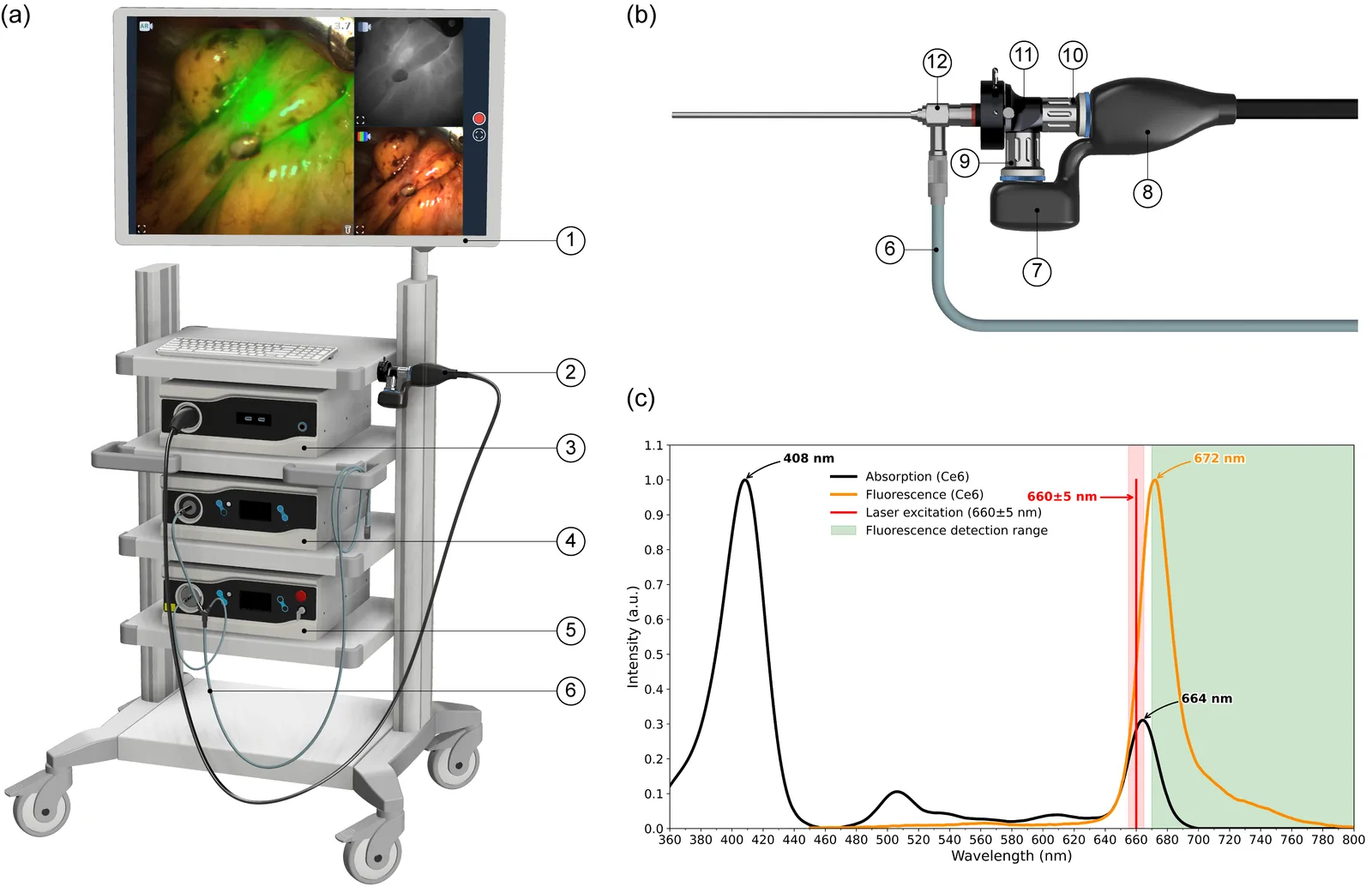
Dual-channel endoscopic video system VENERA-red-660: (**a**) General view of the endoscopic system showing the main units (1—monitor; 2—camera unit; 3—data processing unit; 4—white-light source; 5—laser source; 6—optical fiber bundle for delivering laser and white light); (**b**) Detailed view of the camera unit showing the main components (7—color camera; 8—monochrome camera; 9—varifocal lens of the color camera; 10—varifocal lens of the monochrome camera; 11—adapter with dichroic beamsplitter; 12—endoscope); (**c**) Absorption and fluorescence spectra of Ce6 indicating laser excitation (red-shaded band) and fluorescence detection ranges (Photolon^®^ dissolved in human blood plasma at a concentration of 10 mg/L).

For intraoperative fluorescence visualization of sensitized tissue boundaries and quantitative assessment of PS distribution, the dual-channel video-fluorescence system VENERA-red-660 (BIOSPEC Ltd., Moscow, Russia) was employed. The system comprises the following main components: a broadband white-light source, a 660 nm semiconductor laser, a Y-shaped fiber bundle, an endoscopic coupler compatible with a standard endoscope, and two recording CMOS cameras, a high-sensitivity monochrome and a color camera, for the simultaneous detection of backscattered light and fluorescence emission (Figure 2). The 660 nm semiconductor laser has a narrow linewidth (FWHM = 0.5 nm) and is additionally filtered by a single-band 660 nm bandpass filter prior to fiber coupling. The excitation and fluorescence channels are separated using a dichroic beamsplitter and a longpass emission filter with a cutoff of 670 nm, which is placed in front of the monochrome camera to suppress residual excitation light in the fluorescence detection path.

The optical characteristics of this system are optimal for fluorescence visualization using chlorin-based PSs and methylene blue. The system provides image acquisition in three imaging modes: color, monochrome, and overlay. In color (navigation) mode, standard white-light endoscopic visualization is performed using a high-sensitivity CMOS camera (Figure 2b). In monochrome mode, the fluorescence signal is recorded by a high-sensitivity monochrome CMOS camera. In overlay (fluorescence) mode, the software provides hardware synchronization of both cameras, implemented via a software trigger initiating simultaneous frame capture on both sensors; the measured temporal offset between channels did not exceed 600 μs, which, given the exposure times used (≥15 ms), does not introduce motion artifacts during endoscopic imaging, and generates a real-time composite image, in which fluorescence regions at wavelengths above 670 nm are highlighted using green pseudo-coloring. This mode allows for the simultaneous evaluation of the anatomical view and the spatial distribution of the PS within the target tissues without interrupting the procedure.

For intraoperative quantitative assessment of PS accumulation, the system enables the calculation of the fluorescence contrast index (FCI) within the region of interest, defined as *FCI* = *I_tumor_*/*I_normal_*, where *I_tumor_* and *I_normal_* are the mean corrected fluorescence intensities of the tumor and normal (healthy) tissue, respectively. Tumor and normal-tissue regions of interest were selected manually by an experienced physician based on visual inspection and anatomical landmarks. To establish the baseline, the endoscope was directed at a region of normal tissue and a calibration procedure was performed, during which the system recorded and stored this value as *I_normal_*; the same reference region and calibrated baseline were used for comparisons before and after PDT. Prior to intensity calculation, the system performed pixel-wise subtraction of a background frame acquired under dark (no-light) conditions. To compensate for variations in working distance, viewing angle, and tissue geometry across different intraoperative scenarios, reported FCI values were computed from corrected rather than raw camera intensities, using a ratiometric correction normalized to the red channel of the color (RGB) camera. Because FCI is computed as a ratio between tumor and adjacent normal tissue captured within the same image frame, it is inherently normalized against variations in working distance and viewing angle encountered across different intraoperative access routes. Regions with FCI > 1 a.u. are defined as areas with elevated PS accumulation.

### 2.2. Photosensitizers

For photodynamic diagnosis and PDT, commercially available and clinically approved chlorin-based PSs were used, including Fotoran E6^®^ (RANPHARMA, Moscow, Russia) and Photolon^®^ (BELMEDPREPARATY, Minsk, Belarus). The PS was dissolved at a dosage of 1 mg/kg of patient body weight in 150 mL of 0.9% sodium chloride solution and administered to all patients by intravenous infusion over 30 min, 3 to 3.5 h prior to fluorescence diagnosis and PDT. Administration was performed in a darkened room to prevent premature photoactivation of the PS. For 24 h following PS administration, patients strictly adhered to light protection protocols. During the first three days after PS administration, exposed areas of the skin were treated with photoprotective cream to prevent phototoxic reactions.

### 2.3. Tissue-Simulating Optical Phantoms

The sensitivity of the system to the Ce6 fluorescence signal was evaluated by imaging aqueous samples containing various concentrations of the PS (Photolon^®^). Intralipid 20% MCT/LCT, widely used as a scattering agent in optical phantoms, served as the light-scattering medium. To simulate the scattering properties of human skin, the lipid emulsion was diluted with distilled water to a concentration of 1.6%, at which the reduced scattering coefficient in the 630–665 nm range is 1.8–2.5 mm^−1^, corresponding to the optical properties of skin in vivo [30]. The concentrations of Photolon^®^ in the phantoms were 0.1, 0.25, 0.5, 1, 2, and 5 mg/L, corresponding to the range of Ce6 concentrations observed in tumor tissue [31]. Images were acquired at a distance of 5 cm while maintaining a constant camera exposure time. The power density of 660 nm laser radiation on the surface of optical phantoms during video-fluorescence imaging was 10–12 mW/cm^2^. At each Ce6 concentration, FCI was measured in 10 repeated acquisitions, with a mean coefficient of variation of 10% across the tested concentration range.

### 2.4. Spectral Fluorescence Diagnostics

For local spectral fluorescence diagnostics, a LESA-01-BIOSPEC laser electron-spectroscopic system (BIOSPEC Ltd., Moscow, Russia) was employed. This system consists of the following main components: a He-Ne laser source (λex = 632.8 nm), a portable spectrometer, a diagnostic probe, and specialized UnoMomento software (version 1.0.6.0, BIOSPEC Ltd., Moscow, Russia) for processing recorded spectral data with normalization to the instrument response function of the system [32]. Spectral signal acquisition was performed in the wavelength range from 600 to 850 nm. The diagnostic probe incorporates six collection optical fibers positioned around a central delivery optical fiber. The core diameter of each optical fiber is 200 µm, with a numerical aperture of NA = 0.22. This probe configuration allows for varying the distance between the probe tip and the biological tissue surface from 0 to 20 mm, as well as the tilt angle from 0° to 30°, with a measurement error not exceeding 10%.

### 2.5. Confocal Microscopy

Microscopic evaluation of Ce6 distribution in peritoneal metastatic lesions of patients with PC was performed using confocal microscopy. During the biopsy procedure, radical surgical resection of the tumor was not performed; targeted biopsy was utilized to obtain tissue samples. The size of each biopsy specimen did not exceed 3 × 3 × 3 mm. Following collection, specimens were placed in 0.9% isotonic NaCl solution and examined within 24 h. Prior to microscopic examination, the biopsy specimens were embedded in Neg-50 freezing medium (Richard-Allan Scientific, Kalamazoo, MI, USA) and frozen until completely solidified. Cryostat sections with a thickness of 20 µm were prepared using a Microm HM 540 cryostat (Thermo Fisher Scientific Microm International GmbH, Walldorf, Germany). Sections were mounted on microscope slides, coverslipped with glycerol, and examined without additional staining. Spectral fluorescence analysis was performed using an LSM 710 NLO laser scanning confocal microscope (Carl Zeiss Microscopy, Oberkochen, Germany). Tissue autofluorescence was excited using a 488 nm laser, whereas Ce6 fluorescence was excited using a 633 nm laser. Plan-Apochromat 20×/0.8 and 63×/1.4 oil immersion objectives were used for visualization.

### 2.6. Photodynamic Therapy

PDT was performed using the same 660 nm laser source integrated into the VENERA-red-660 system (maximum power output 2000 mW) that was also used for Ce6 fluorescence excitation (Section 2.1), consistent with the single-source phototheranostic concept described above. The criterion for completing a PDT session was achieving a PS photobleaching level exceeding 50%, consistent with our group’s prior work on photobleaching-guided PDT personalization using the same 660 nm excitation approach [9,31,33]. Irradiation was terminated adaptively based on real-time FCI monitoring once this threshold was exceeded, rather than delivering a pre-prescribed fixed light dose; accordingly, the light-dose ranges reported below reflect the range of doses actually delivered across patients rather than a protocol-fixed value. Laser power density and total light dose varied depending on the lesion site and are detailed below.

#### 2.6.1. Photodynamic Therapy of Basal Cell Carcinoma

A flat-cut quartz optical fiber with a core diameter of 600 µm and NA = 0.22 was used for laser delivery. The laser power density ranged 350–400 mW/cm^2^. The total light dose was 150–180 J/cm^2^. Non-uniformity of laser illumination on the tumor surface did not exceed 10% due to a microlens attached to the distal end of the fiber.

#### 2.6.2. Photodynamic Therapy of Vulvar Intraepithelial Neoplasia

A flat-cut quartz optical fiber with a core diameter of 600 µm and NA = 0.22 was used for laser delivery. The laser power density was approximately 300–350 mW/cm^2^. The total light dose was approximately 150 J/cm^2^. Non-uniformity of laser illumination on the tumor surface did not exceed 10% due to a microlens attached to the distal end of the fiber.

#### 2.6.3. Photodynamic Therapy of Peritoneal Carcinomatosis

A polymer optical fiber with a 20 mm long cylindrical diffuser was used for laser light delivery. The laser power density was 50–150 mW/cm^2^. The total light dose was 10–50 J/cm^2^.

### 2.7. Patients and Clinical Protocol

The study included 18 patients aged 35 to 75 years with morphologically verified neoplasms across three localizations: BCC (*n* = 5), VIN III (*n* = 3), and PC (*n* = 10) secondary to recurrent high-grade serous ovarian carcinoma (HGSOC). Group-specific inclusion and exclusion criteria are summarized in Table 1.

The clinical protocol comprised several sequential stages:

1. Video fluorescence imaging to delineate sensitized tumor boundaries prior to PDT;
2. Spectral fluorescence diagnostics to verify PS accumulation prior to PDT;
3. PDT of the tumor lesion;
4. Video fluorescence imaging to evaluate PS photobleaching after PDT;
5. Spectral fluorescence diagnostics to verify PS photobleaching after PDT.

All patients in the PC group had a history of HGSOC treated with primary or interval cytoreductive surgery followed by systemic chemotherapy. At the time of study enrollment, disease recurrence had presented as peritoneal carcinomatosis, and it was this recurrent peritoneal disease—rather than the primary ovarian tumor—that was targeted by the phototheranostic protocol described here. Treatment was administered using a combined approach that included laparoscopy and pressurized intraperitoneal aerosol chemotherapy (PIPAC) in combination with PDT alongside systemic chemotherapy. Systemic chemotherapy was prescribed according to one of the following regimens: gemcitabine + cisplatin + bevacizumab or gemcitabine + docetaxel + bevacizumab. The combined treatment was carried out according to the following schedule: laparoscopy with PIPAC and PDT was performed after every two cycles of systemic chemotherapy. In total, each patient underwent three cycles of PIPAC and PDT alternating with cycles of systemic chemotherapy. During each surgical intervention, a peritoneal biopsy was performed, followed by histological examination and assessment of the pathomorphological response.

### 2.8. Statistical Analysis

Statistical data processing and visualization were performed using the Python 3 programming language (utilizing the pandas library for data structuring and SciPy for computations). Patient-level statistical analysis was performed specifically for the FCI, the primary quantitative endpoint of this study. For each patient, FCI measurements were averaged to obtain a single patient-level value before and after PDT; statistical comparisons were therefore performed at the patient level (*n* = 5 for BCC, *n* = 3 for VIN III, *n* = 10 for PC), with each patient constituting one independent observation. The statistical significance of differences between pre- and post-PDT FCI values was assessed using the paired Student’s *t*-test; normality of the paired differences was confirmed using the Shapiro–Wilk test. The threshold for statistical significance was set at *p* < 0.05. The complementary spectral fluorescence and backscatter measurements obtained independently using the LESA-01-BIOSPEC system were acquired from a representative patient in each group and are reported descriptively (mean ± SD across repeated measurements within that patient) as qualitative confirmation of the FCI-based findings; they were not subjected to patient-level inferential statistical testing.

## 3. Results

### 3.1. System Sensitivity Assessment on Optical Phantoms

**Figure 3:**
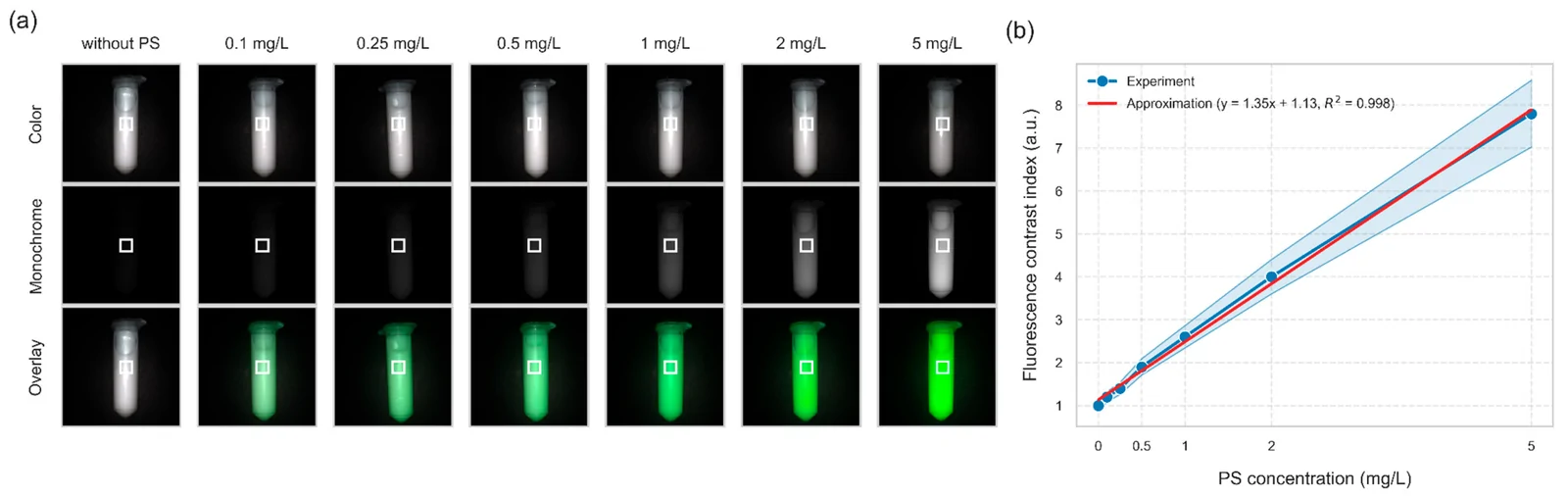
Sensitivity evaluation of the VENERA-red-660 system: (**a**) Color, monochrome (fluorescence), and overlay images of Ce6 in aqueous solution at concentrations of 0.1, 0.25, 0.5, 1, 2, and 5 mg/L. (**b**) FCI as a function of Ce6 concentration in optical phantoms. FCI was calculated within the marker-selected regions of the images, averaged over 10 repeated acquisitions per concentration. The shaded band represents the variability (±10% SD) of FCI across these repeated measurements.

To evaluate PS detection sensitivity and assess the linearity of the fluorescence response of the dual-channel VENERA-red-660 system, a series of experiments was conducted on liquid optical phantoms simulating the light-scattering properties of human soft tissues in the spectral range of 630 to 665 nm. The concentration of the chlorin-based PS (Photolon^®^) ranged from 0.1 to 5.0 mg/L. Figure 3a displays the acquired images across three imaging modes: color, monochrome, and overlay (in which the fluorescence signal is highlighted in green over the color image). Notably, the color and fluorescence channels are precisely co-registered with no visible spatial displacement, confirming the accurate hardware synchronization of the two CMOS cameras. For the phantom measurements, FCI was defined in the same way as in the clinical definition: as the ratio of the fluorescence intensity of each Ce6-containing sample to the fluorescence intensity of the Intralipid phantom without PS shown in Figure 3a. This phantom served as the reference (FCI = 1 a.u.), similar to the role of adjacent normal tissue as the reference in the clinical measurements.

The obtained results demonstrate high sensitivity in detecting Ce6 fluorescence under 660 nm laser excitation. The lowest Ce6 concentration included in this study, 0.1 mg/L, remained clearly distinguishable from the PS-free background at working distances up to 5 cm—a level well below the PS concentrations typically detected in tumor tissues in vivo [31]. A formal limit of detection was not calculated in this study. Over the evaluated concentration range of 0.1 to 5.0 mg/L, the fluorescence response of Ce6, expressed as FCI, demonstrated a strictly linear dependence on PS concentration (R^2^ = 0.998) (Figure 3b). The existence of a direct linear relationship between FCI and PS concentration confirms the feasibility of accurate quantitative assessment of spatial drug distribution in optical phantoms, supporting its potential applicability during future clinical measurements. However, this linear phantom response does not by itself establish quantitative accuracy of Ce6 concentration measurements in vivo, where tissue absorption, scattering, illumination geometry, and collection efficiency can vary substantially from the controlled phantom conditions. The present study did not attempt to quantify absolute Ce6 tissue concentration; FCI was used only as a relative comparison between tumor and adjacent normal tissue. Extending this phantom-based calibration to absolute in vivo concentration measurements would require accounting for tissue-specific optical properties and remains a direction for future work.

### 3.2. Clinical Evaluation of Fluorescence Diagnostics Efficacy

Initially, the study was conducted on tumors at visually accessible anatomical sites: BCC (*n* = 5) and VIN III (*n* = 3) via external access. Following optimization of the methodology, the phototheranostic approach was applied in patients with PC in combination with PIPAC (*n* = 10) during laparoscopic procedures. All procedures were performed routinely without complications; no systemic adverse events or unexpected cutaneous phototoxicity were recorded.

#### 3.2.1. Fluorescence Diagnostics During Photodynamic Therapy of Basal Cell Carcinoma

**Figure 4:**
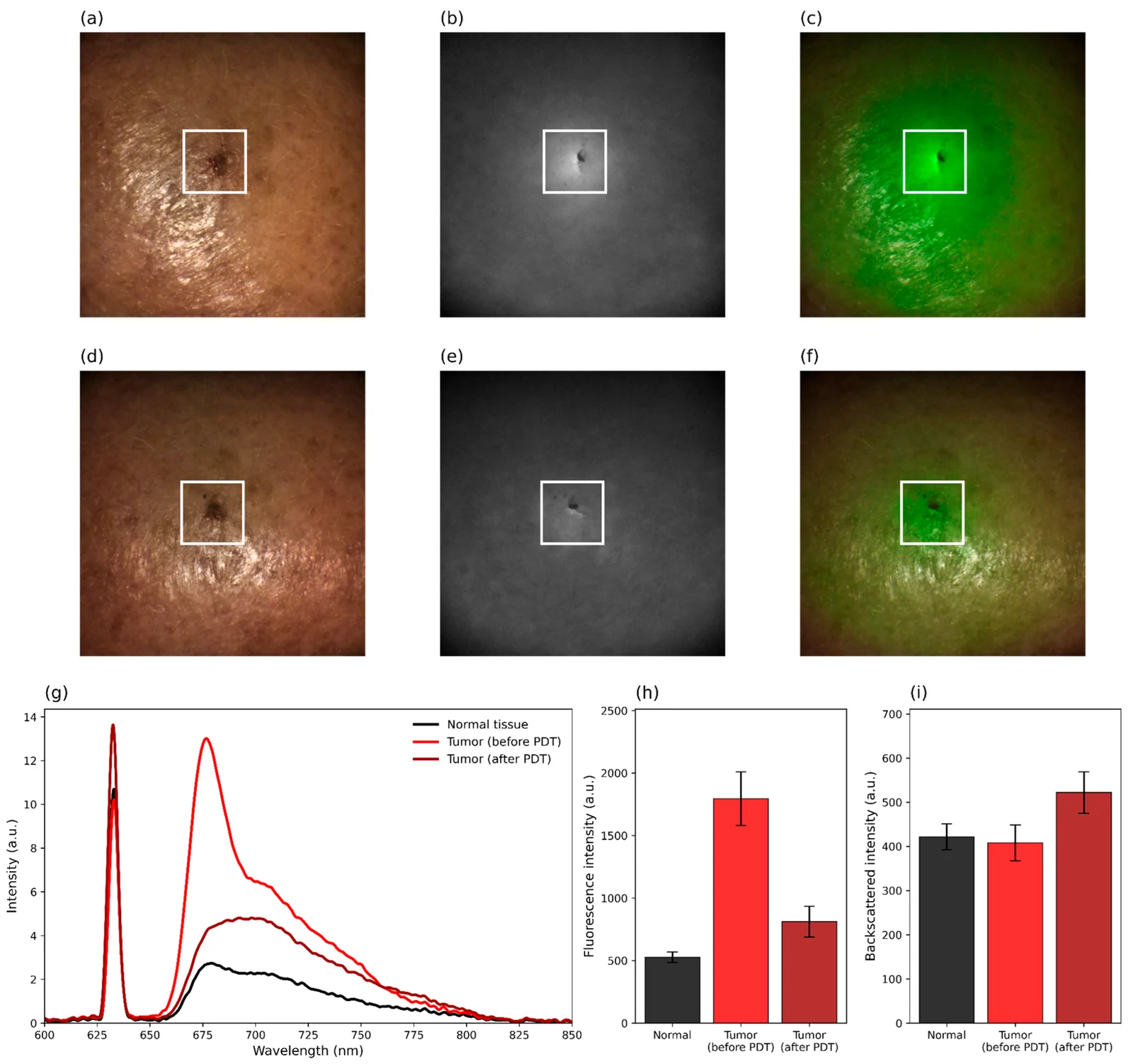
Fluorescence navigation of Ce6 accumulation in BCC: (**a**–**f**) Video fluorescence imaging obtained with the VENERA-red-660 system: (**a**–**c**) before PDT, (**d**–**f**) immediately after PDT. (**g**–**i**) Spectral fluorescence diagnostic results obtained independently using the LESA-01-BIOSPEC spectroscopic system: (**g**) Recorded spectral data of normal tissue and tumor before and after PDT, including backscattered laser radiation (λ_ex_ = 632.8 nm) and tissue fluorescence in the range of 650–850 nm. (**h**) Integrated fluorescence intensity in the range of 665–695 nm. (**i**) Integrated backscattered laser radiation intensity in the range of 625–640 nm. Scale bar: 10 mm. The square marker highlights the region where FCI was calculated.

During fluorescence diagnostics in patients with skin BCC, the VENERA-red-660 system in overlay mode provided clear visualization of tumor boundaries against surrounding normal tissues. Prior to PDT, a prominent fluorescence signal corresponding to Ce6 accumulation, highlighted in green pseudo-color, was observed in tumor lesions, whereas surrounding normal tissues exhibited a significantly lower fluorescence response (Figure 4a–c). The FCI prior to PDT was 3.0 ± 0.4 a.u. (*n* = 5), indicating pronounced selective accumulation of Ce6 in the tumor relative to normal tissues. Following completion of the PDT session, the fluorescence signal intensity within the tumor lesion decreased significantly (Figure 4d–f), and the FCI dropped by 57% to 1.3 ± 0.2 a.u. (paired *t*-test, t(4) = 11.76, *p* < 0.001), reflecting PS photobleaching during photodynamic exposure and satisfying the predefined session completion criterion (photobleaching > 50%).

Obtained independently using the separate LESA-01-BIOSPEC spectroscopic system in a representative patient from this group, spectral fluorescence diagnostic data showed a characteristic Ce6 fluorescence peak in the region of 665–695 nm in areas of tumor infiltration and normal tissue (Figure 4g) [31]. Prior to PDT, the integrated tissue fluorescence intensity in the range of 665–695 nm was 526 ± 42 a.u. for normal tissue and 1795 ± 215 a.u. for tumor tissue (mean ± SD across repeated measurements within this patient) (Figure 4h). Following PDT, the tumor fluorescence intensity decreased by 55% to 811 ± 122 a.u. Concurrently, the intensity of backscattered diagnostic laser radiation in the range of 625–640 nm was analyzed; after PDT, the intensity in the tumor increased by 28%, from 408 ± 41 to 522 ± 47 a.u. (Figure 4i). This increase is consistent with PDT-induced changes in tumor optical properties and vascular response and aligns with previously published data on the vascular effects of PDT [9,31,32,34].

#### 3.2.2. Fluorescence Diagnostics During Photodynamic Therapy of Vulvar Intraepithelial Neoplasia

**Figure 5:**
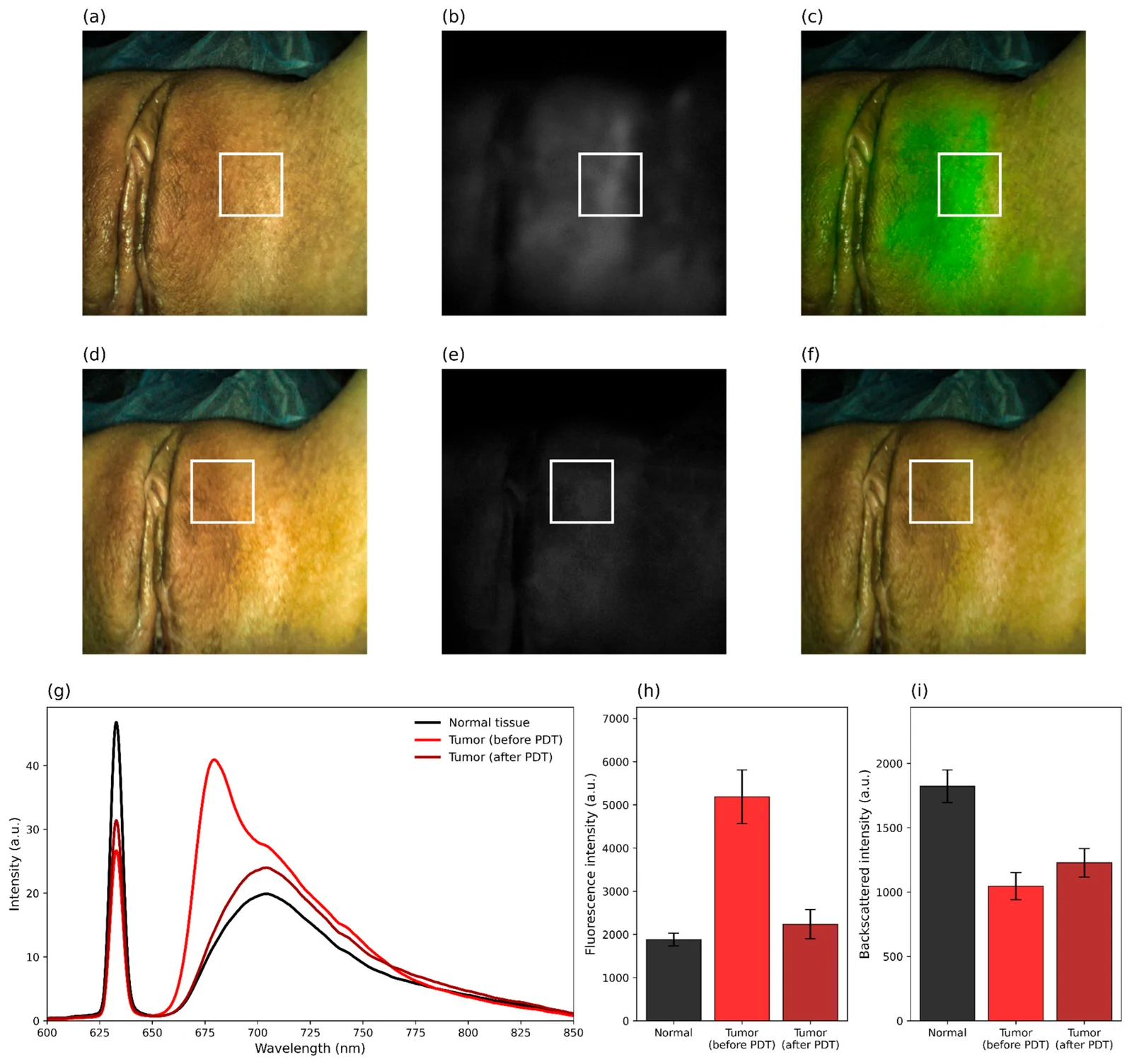
Fluorescence navigation of Ce6 accumulation in VIN III: (**a**–**f**) Video fluorescence imaging obtained with the VENERA-red-660 system: (**a**–**c**) before PDT, (**d**–**f**) after PDT. (**g**–**i**) Spectral fluorescence diagnostic results obtained independently using the LESA-01-BIOSPEC spectroscopic system: (**g**) recorded spectral data of normal tissue and tumor before and after PDT, including backscattered laser radiation (λ_ex_ = 632.8 nm) and tissue fluorescence in the range of 650–850 nm. (**h**) Integrated fluorescence intensity in the range of 665–695 nm. (**i**) Integrated backscattered laser radiation intensity in the range of 625–640 nm. Scale bar: 10 mm. The square marker highlights the region where FCI was calculated.

During fluorescence diagnostics in patients with VIN III, the VENERA-red-660 system in overlay mode provided visualization of neoplastic lesions via external access using a rigid adapter. Prior to PDT, a prominent Ce6 fluorescence signal was recorded in the lesion area, and the FCI was 3.1 ± 0.2 a.u. (*n* = 3) (Figure 5a–c).

Following completion of the PDT session, the fluorescence of the tumor lesion decreased (Figure 5d–f), and the FCI dropped by 66% to 1.0 ± 0.3 a.u. (paired *t*-test, t(2) = 10.03, *p* = 0.010). Obtained independently using the separate LESA-01-BIOSPEC spectroscopic system in a representative patient from this group, the integrated Ce6 fluorescence intensity in normal tissue prior to PDT in the region of 665–695 nm was 1880 ± 150 a.u., and in tumor tissue it was 5186 ± 622 a.u. (mean ± SD across repeated measurements within this patient) (Figure 5g–h). Following PDT, the tumor fluorescence intensity decreased by 57% to 2237 ± 336 a.u., which also confirms Ce6 photobleaching (Figure 5h). The intensity of backscattered laser radiation in the tumor after PDT increased from 1046 ± 105 to 1227 ± 111 a.u. (Figure 5i), which is consistent with PDT-induced changes in tissue optical properties and vascular response.

#### 3.2.3. Fluorescence Diagnostics During Photodynamic Therapy of Peritoneal Carcinomatosis

**Figure 6:**
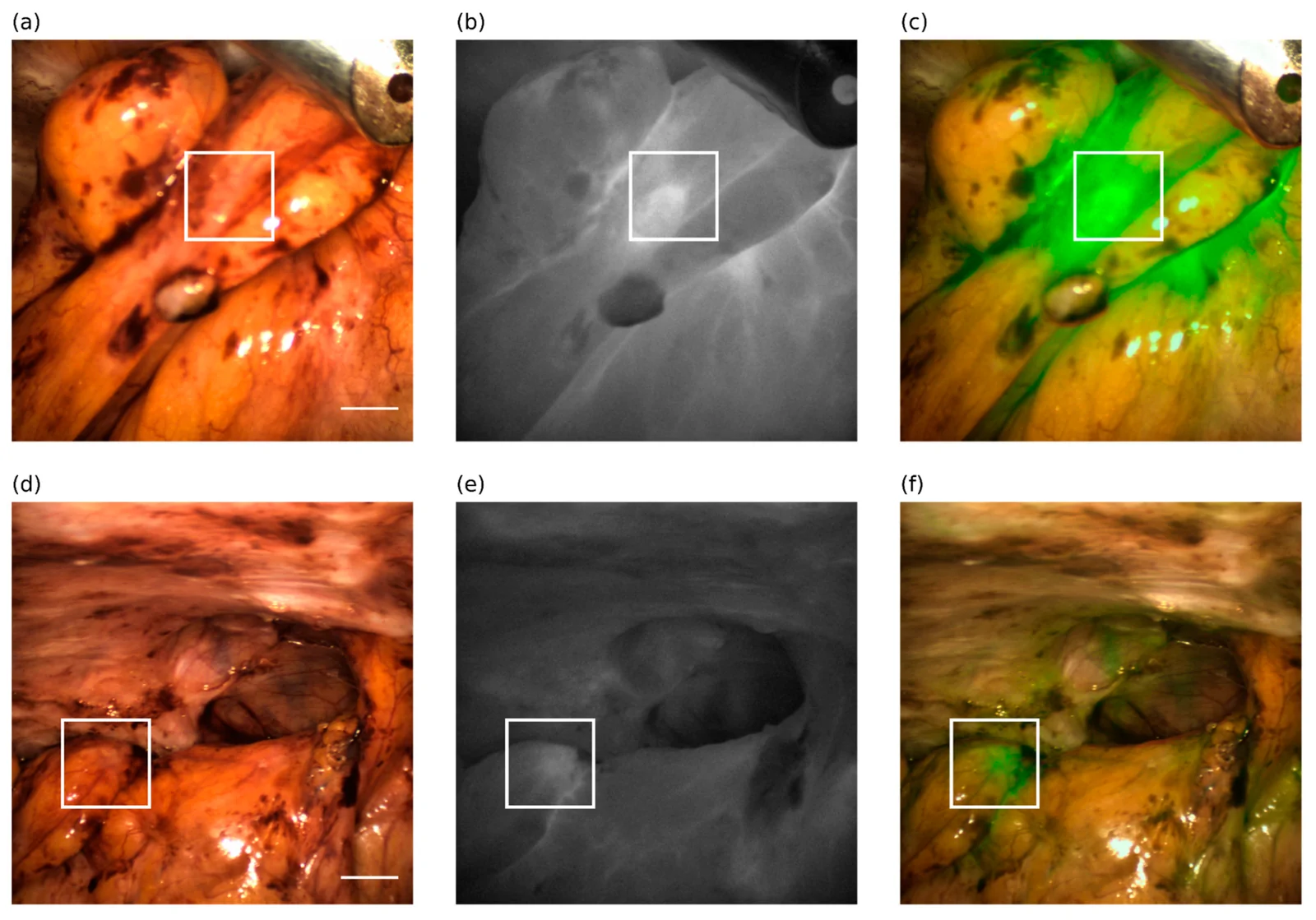
Video fluorescence imaging of Ce6 accumulation in metastatic sites in PC, obtained with the VENERA-red-660 system: (**a**–**c**) Before PDT. (**d**–**f**) Immediately after PDT. Scale bar: 10 mm. The square marker highlights the region where FCI was calculated.

During intraoperative fluorescence diagnostics during laparoscopic surgery in patients with PC, the VENERA-red-660 system enabled visualization of Ce6-accumulating metastatic foci against surrounding unaltered peritoneal tissues with high fluorescence contrast (Figure 6a–c). Prior to PDT, the FCI in metastatic sites was 3.8 ± 0.4 a.u. (*n* = 10), representing the highest value among all three studied nosological groups, which reflects the high selectivity of Ce6 accumulation in peritoneal metastatic lesions. Following completion of PDT, the FCI decreased by 60% to 1.5 ± 0.3 a.u. (paired *t*-test, t(9) = 17.46, *p* < 0.0001), confirming effective Ce6 photobleaching and satisfying the session completion criterion (Figure 6d–f).

**Figure 7:**
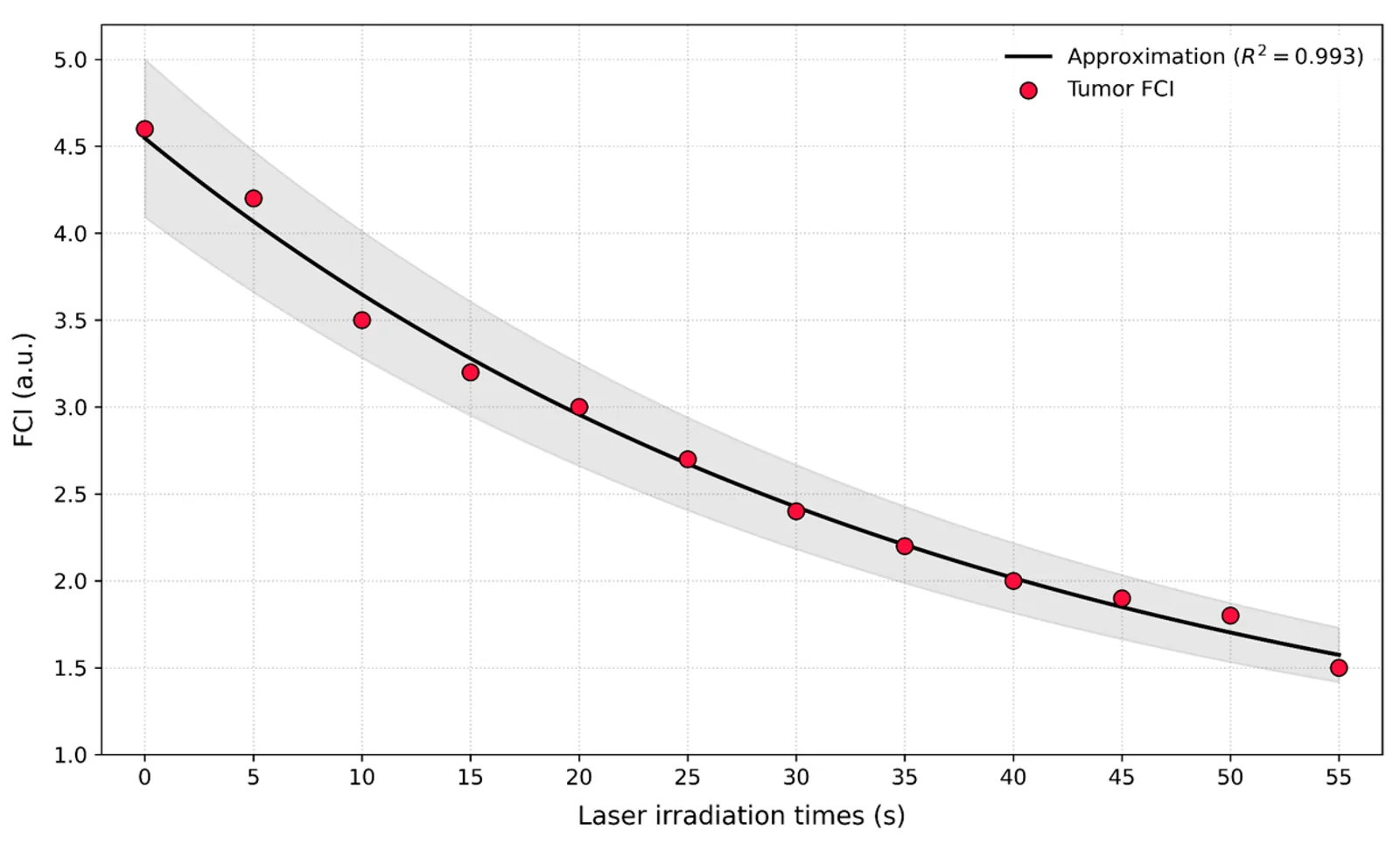
Real-time dynamics of tumor FCI during a single PDT exposure in a representative patient from the PC group. Red circles indicate tumor FCI values recorded at 5 s intervals over 55 s of continuous 660 nm laser irradiation; the black line shows the fitted exponential decay (R^2^ = 0.993), and the shaded band represents ±10% measurement variability of VENERA-red-660 FCI determination, based on the reproducibility of repeated measurements on tissue-simulating optical phantoms.

To determine whether the FCI changes observed between pre- and post-PDT time points reflect continuous photobleaching kinetics, real-time fluorescence contrast monitoring was performed throughout laser irradiation in a pilot subset of two patients. Throughout this monitoring, the 660 nm laser remained continuously on at full therapeutic irradiance, with no shuttering, modulation, source switching, or frame interleaving; fluorescence and backscattered-light signals were acquired concurrently via the dual-camera configuration described in Section 2.1, confirming genuinely simultaneous, rather than rapidly alternating, diagnostic and therapeutic operation. Figure 7 shows the corresponding dynamics for a representative PC lesion, in which tumor FCI was recorded at 5 s intervals over the full duration of a single PDT exposure.

**Figure 8:**
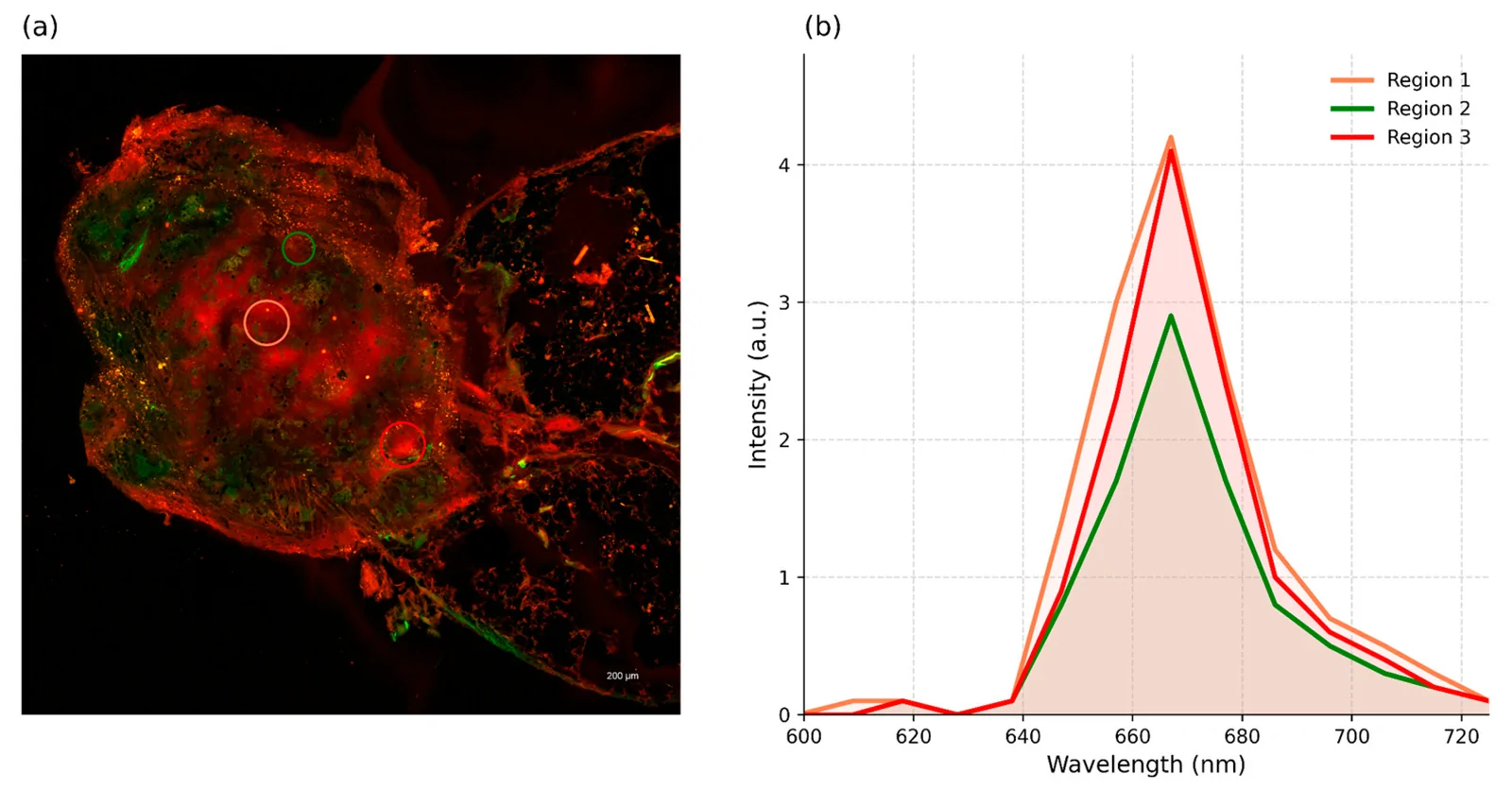
Confocal microscopy of a tumor biopsy from a patient with PC 3 h after Ce6 administration: (**a**) Fluorescence image of the tumor. (**b**) Fluorescence spectra of the tumor in marker-highlighted zones. Green represents autofluorescence intensity in the range of 500–620 nm; red represents Ce6 fluorescence intensity in the range of 640–725 nm.

For morphological verification of the selectivity of Ce6 accumulation, a targeted biopsy of a metastatic lesion was obtained from one patient 3 h after intravenous Ce6 administration prior to the initiation of PDT. The resulting biopsy specimens were examined using confocal fluorescence microscopy (Figure 8).

In fluorescence images, the green channel (500–620 nm) reflects tissue autofluorescence intensity, while the red channel (640–725 nm) corresponds to Ce6 fluorescence (Figure 8a). In regions of tumor infiltration, the signal in the red channel exceeded the autofluorescence signal. Spectral analysis confirmed the presence of a characteristic Ce6 fluorescence peak in the region of 660–670 nm (Figure 8b). The obtained findings at the cellular level verify the selective accumulation of Ce6 in peritoneal metastatic lesions and confirm the specificity of the fluorescence signal recorded by the VENERA-red-660 system in vivo.

## 4. Discussion

In this study, we present the initial results of the clinical evaluation of a novel dual-channel video fluorescence endoscopic system, VENERA-red-660, for intraoperative fluorescence diagnostics during photodynamic diagnosis and PDT in patients with cutaneous BCC, PC, and VIN III using chlorin-based PSs, namely Fotoran E6^®^ or Photolon^®^. At present, several chlorin-based PSs approved for clinical use are known, with their main properties summarized in Table 2.

It should be noted that the absorption and fluorescence properties of chlorin-based PSs can vary depending on the microenvironment and PS concentration in the medium (Table 2). According to our data, Photolon dissolved in blood plasma exhibits two prominent absorption peaks: in the Soret band at 408 nm and in the red spectral region with a maximum at 664 nm (Figure 2c). The fluorescence peak of Photolon^®^ upon excitation in the Soret band is observed at 672 nm. The presence of two absorption peaks in Ce6 provides a fundamental opportunity to induce fluorescence excitation and deliver a therapeutic effect in both the blue (~403 to 420 nm) and red (~650 to 665 nm) spectral regions (Table 2); however, the physical implications of this choice differ fundamentally. Although the absorption coefficient of Ce6 at the Soret peak is nearly 15 times higher than in the Q-band (660 nm), the in vivo efficacy of fluorescence diagnostics is limited by the optical properties of the biological tissue itself.

Radiation with a wavelength of 405 nm falls within the Soret band of heme-containing proteins, located in the 400–440 nm range [38]. Analysis of the absorption coefficients of *Hb* and *HbO_2_* demonstrates that the absorption intensity in the Soret region (λ = 405 nm) exceeds the corresponding value in the red spectral region (λ = 660 nm) by more than 215 times [39]. Hemoglobin acts as an “optical barrier” capable of almost completely absorbing probing light in superficial layers, thereby preventing the visualization of deep-seated tumor structures. An additional factor limiting light penetration depth is scattering, as the transition from the blue to the red spectral range is accompanied by a significant reduction in the tissue scattering coefficient [40,41].

Upon excitation with 405 nm light, the therapeutic effect is realized predominantly in superficial tissue layers at a depth of up to 0.5 mm, whereas the use of a wavelength of 660 nm provides therapeutic impact to a depth of approximately 2 mm [42,43,44,45]. Experimental and clinical studies confirm that Ce6-mediated PDT with excitation at 405 nm is most effective in treating superficial lesions, which aligns with morphological analysis results and calculated data regarding absorbed energy distribution in tissue [43]. Conversely, when using 660 nm laser radiation, the greater penetration depth allows the therapeutic effect to encompass the entire tumor volume, including the perifocal zone of tumor invasion, which is of fundamental importance.

The increase in backscattered diagnostic radiation observed after PDT in the BCC and VIN III groups may reflect PDT-induced changes in tissue optical properties and microcirculation. However, because this signal is jointly determined by tissue absorption and scattering, it should be considered an indirect marker of the vascular response rather than direct evidence of thrombosis. Optical spectral methods capable of separately assessing tissue oxygen saturation and relative blood content have previously been used to monitor tissue response to 660 nm PDT [46] and to evaluate tissue blood supply in vivo [47]. These same PDT-induced changes in tissue absorption and scattering could also influence the measured fluorescence signal independently of true photosensitizer photobleaching. To address this issue, VENERA-red-660 applies a real-time ratiometric correction to all reported FCI values, normalized to the red channel of the color camera, which captures backscattered 660 nm light concurrently with the fluorescence signal (Section 2.1). It compensates for variations in tissue optical properties in addition to working distance and viewing angle. This correction is empirical rather than a full physical model of tissue absorption and scattering, so a residual contribution of changes in optical properties to the decrease in FCI cannot be fully excluded, and the observed decrease is therefore interpreted as being consistent with, but not necessarily exclusively attributable to, photobleaching.

A key feature of our system is the capability for PS fluorescence excitation at a wavelength of 660 ± 5 nm, allowing diagnostics to be conducted without interrupting the PDT session, thereby realizing phototheranostics. The complexity of fluorescence imaging at λ_ex_ = 660 nm during Ce6-mediated PDT is primarily attributed to the small Stokes shift in chlorin-based PSs, which is only approximately 2–8 nm. Additionally, the comparable bandwidth of therapeutic laser and light-emitting diode systems must be taken into consideration. The adequacy of excitation-light rejection was empirically confirmed during actual PDT sessions at full therapeutic irradiance: the continuous FCI monitoring shown in Figure 7 exhibits a smooth, artifact-free exponential decay, indicating no detectable excitation leakage or camera saturation under these conditions.

Despite the clear advantages of fluorescence diagnostics, its integration into PDT presents several significant technical challenges. Currently available endoscopic systems generally do not support simultaneous operation in white light and fluorescence imaging modes, forcing the clinician to interrupt the procedure to assess PS distribution or its photobleaching intensity [48]. This limitation is illustrated by a bronchoscopic autofluorescence system, which required switching away from the therapeutic beam for diagnostic imaging and still produced a false-negative fluorescence signal after photosensitizer administration; a complementary “simultaneous-type” video-endoscope reported in the same clinical series avoided laser-induced video white-out but was not designed for fluorescence-based PS detection at all [49]. This complicates the treatment workflow and reduces the precision of light dose monitoring, impairing intraoperative navigation and potentially compromising both the efficacy and safety of PDT. To address this limitation, previous attempts have been made to develop combined video systems that merge white light and fluorescence imaging modalities [10,12,50,51]. Table 3 provides a comparison between the VENERA-red-660 system and leading endoscopic video fluorescence platforms utilized in clinical practice for photodynamic diagnosis.

**Table 3:** Comparative characteristics of endoscopic fluorescence imaging systems for photodynamic diagnosis.

System	Company	Photosensitizer	Excitation Light	Emission Filters	Quantitative Assessment	Reference
D-Light C	Karl Storz, Tuttlingen, Germany	5-ALA/HAL/MAL	375–445 nm	600–740 nm	No	[52]
HD-Xoscope	Karl Storz, Tuttlingen, Germany	5-ALA/HAL/MAL	380–450 nm	n.a.	No	[53]
Blue Light Cystoscopy	Karl Storz, Tuttlingen, Germany	5-ALA/HAL/MAL	380–450 nm	n.a.	No	[54]
SAPHIRA BLI-system	Karl Storz, Tuttlingen, Germany	5-ALA/HAL/MAL	380–420 nm	n.a.	No	[55]
VISERA ELITE II	OLYMPUS, Tokyo, Japan	5-ALA/HAL/MAL	400–410 nm	n.a.	No	[55]
System blue	RICHARD-WOLF, Vernon Hills, IL, USA	5-ALA/HAL/MAL	380–500 nm	n.a.	No	[55]
Modified system: PINPOINT + Aladuck LS-DLED	Stryker, Portage, MI, USA + SBI Pharmaceuticals, Tokyo, Japan	5-ALA/HAL/MAL	400–410 nm	n.a.	No	[56]
SAFE-3000	Pentax, Tokyo, Japan	5-ALA/HAL/MAL	408 nm	n.a.	No	[57]
Fiberscope N-4L	Machida, Abiko, Chiba, Japan	5-ALA/HAL/MAL	405 nm	n.a.	No	[58]
ELUXEO 7000 endoscopy system	FUJIFILM, Tokyo, Japan	Chlorin-based PSs	410 ± 10 nm	n.a.	No	[59]
Endoscopic video system/VENERa-red	BIOSPEC Ltd., Moscow, Russia	Chlorin-based PSs, 5-ALA/HAL/MAL	635 nm	>660 nm	Yes	[10,17]
Laser video endoscopy system	Custom, Saint Petersburg, Russia	Chlorin-based PSs, ICG	405 nm, 808 nm	n.a.	No	[12]
VENERA -red-660	BIOSPEC Ltd., Moscow, Russia	Chlorin-based PSs	660 ± 5 nm	>670 nm	Yes	This study

Abbreviations: 5-ALA—5-aminolevulinic acid; HAL—hexaminolevulinate; MAL—methylaminolevulinate; ICG—indocyanine green.

As demonstrated in Table 3, VENERA-red-660 is currently the only clinically evaluated endoscopic platform combining laser excitation at 660 nm for photodynamic diagnosis utilizing chlorin-based PSs. The continuous dual-channel operation of the system provides quantitative monitoring of fluorescence intensity and enables compatibility with three types of surgical access within a single PDT protocol.

### 4.1. Photodynamic Diagnosis and Therapy of Basal Cell Carcinoma

PDT is a well-established organ preserving treatment modality for BCC, particularly for superficial and nodular subtypes located in functionally and cosmetically significant areas [60]. A large-scale clinical study involving 1782 patients with BCC (tumors ≤ 2 cm, predominantly located on facial skin) demonstrated that PDT with intravenous administration of Fotoran E6^®^ at a dose of 1.0 mg/kg followed by laser irradiation (670 nm, 300 to 350 mW/cm^2^) achieved complete tumor regression in all patients one month post treatment, with a recurrence rate of 1% at one year and 8.5% over a five year follow up period [61]. In another large-scale study, the efficacy of Ce6 mediated PDT using the photosensitizers Photodithazine^®^ (0.5 to 1.0 mg/kg, *n* = 72), Photolon^®^ (1.1 to 1.6 mg/kg, *n* = 281), and Fotoran^®^ (1.1 to 1.6 mg/kg, *n* = 179) was evaluated in 532 patients with skin BCC aged 28 to 93 years; irradiation sessions were conducted using a Latus 2 laser system (662 nm) at a power density of 200 to 500 mW/cm^2^ and a light energy dose of 100 to 600 J/cm^2^ [62]. Over a follow up period ranging from 6 months to 5 years, recurrences were recorded in 16.2% of cases; for primary BCC with a lesion size up to 2.0 cm, the recurrence rate was 2.2%, whereas for tumors measuring 2.0 to 4.0 cm, it reached 9.5%. Fluorescence diagnostics served as a key tool for planning and evaluating PDT efficacy in these studies, enabling visualization of tumor margins and monitoring of the PS photobleaching level. According to spectral fluorescence diagnostics in 19 patients with BCC, a pronounced contrast of Ce6 accumulation in tumor tissue compared to normal tissue was observed in all patients: 3 h after intravenous administration of Ce6 at a dose of 1 mg/kg, the PS concentration in normal tissues was 0.18 mg/kg, in the tumor it was 1.26 mg/kg, and in the 5 mm wide peritumoral zone it reached 0.63 mg/kg [31]. These findings align with our measured fluorescence contrast index values (3.0 a.u.) and confirm the fundamental feasibility of fluorescence tumor margin demarcation in BCC using the developed system.

### 4.2. Photodynamic Diagnosis and Therapy of Vulvar Intraepithelial Neoplasia

PDT is considered an organ preserving alternative to surgical treatment of VIN III/carcinoma in situ, particularly relevant in widespread multicentric lesions or in patients seeking to avoid destructive interventions. PDT for cervical and vulvar lesions using the photosensitizers 5-aminolevulinic acid (5-ALA), hexaminolevulinate (HAL), methylaminolevulinate (MAL), Ce6, Photofrin II, and Photogem has shown that PDT stimulates an immune response contributing to human papillomavirus (HPV) elimination and lesion regression without thermal tissue damage; moreover, Ce6, activated in the range of 660 to 670 nm, provides deeper tissue penetration and a more pronounced immunomodulatory effect compared to 5-ALA (630 to 635 nm), making it preferable for extensive and deeply localized lesions [63]. Among current therapeutic options for VIN III/preinvasive vulvar cancer are local excision, laser ablation, topical imiquimod application, vulvectomy, and PDT. PDT attracts attention due to its minimal invasiveness and favorable cosmetic outcomes: in a retrospective study of 93 patients with VIN, the rate of scarring and vulvar anatomical alterations following PDT with 5-ALA (635 nm, 100 J/cm^2^) was 0% compared to 12.8% for laser vaporization and 41.6% for surgical excision, with a complete response achieved in 52% of patients [64]. The technique of simultaneous spectral and video fluorescence diagnostics during Ce6 mediated PDT of cervical intraepithelial neoplasia, a disease closely related to VIN in histological structure and HPV etiology, was successfully applied in 52 patients: following the first course of PDT, lesion regression was achieved in 80.8%, with HPV undetected in 92.3%; after the second course, complete regression was observed in 100% of patients [33]. Our data indicate a high selectivity of Ce6 accumulation in the pathological tissues of patients and align with published findings on the high efficacy of PDT with chlorin-based PSs.

### 4.3. Photodynamic Diagnosis and Therapy of Peritoneal Carcinomatosis

PDT for PC represents a promising yet technically challenging approach due to the necessity of uniform irradiation over extensive peritoneal surfaces and intraoperative dose monitoring [65,66,67]. Fluorescence diagnosis using 5-ALA-induced protoporphyrin IX (PpIX) enables the detection of peritoneal tumor nodules invisible under white light examination and has the potential to become an important component of diagnostic laparoscopy with a minimal incidence of adverse events [65]. In a phase II study of intraperitoneal PDT with Photofrin in 100 patients with PC and sarcomatosis (33 with ovarian cancer, 37 with gastrointestinal tumors, and 30 with sarcomas), a complete response at 6 months according to intent to treat analysis was achieved in 9.1%, 5.4%, and 13.3% of patients, respectively; the authors noted that while the procedure was technically feasible, it did not provide significant objective control of tumor growth, largely due to the low accumulation selectivity of first generation PSs (the median tumor to normal tissue ratio for the intestine was only 1.1 to 2.1) and the heterogeneity of optical properties of peritoneal tissues [68]. Another study evaluating intraoperative PDT (IOPDT) in peritoneal lesions demonstrates the promise of this approach. Sidorov et al. presented a comparative analysis of surgical outcomes in 57 patients with pseudomyxoma peritonei of appendiceal origin: in 32 (56.1%) patients, cytoreductive surgery was supplemented with IOPDT (Photogem/Photosens, 630/672 nm), whereas in 25 (43.9%) patients, hyperthermic intraperitoneal chemotherapy (HIPEC) (cisplatin, 43 to 44 °C, 60 min) was performed [69]. Despite significantly worse completeness of cytoreduction scores in the IOPDT group, the 5-year survival rate was 65.2% compared to 86.6% in the HIPEC group; however, the postoperative complication rate in the IOPDT group was significantly lower (11.1% vs. 23.8%) with zero mortality in this group (0% vs. 12.0% in the HIPEC group), allowing the authors to consider IOPDT as an effective and safer alternative in cases with a high peritoneal cancer index (PCI) and contraindications to HIPEC [69]. Clinical experience with phototheranostics in PC also demonstrates the potential of this approach. Rudakov D. A. et al. (2024) presented a clinical case of a two stage cytoreductive surgical treatment in a patient with peritoneal metastases from ovarian cancer, integrating photodynamic diagnosis (405 nm laser) and PDT (662 nm, Fotoran E6^®^, 2.5 mg/kg) combined with hyperthermic intraperitoneal chemotherapy; intraoperative photodynamic diagnosis enabled the identification of visually undetectable residual tumor foci on the diaphragmatic dome, leading to an expanded diaphragmatic peritonectomy and the achievement of a completeness of cytoreduction score of CC0, while histological examination of carcinomatosis nodules following PDT revealed no viable adenocarcinoma foci in the specimens [70]. The highest fluorescence contrast index value (3.8 a.u.) recorded in our PC group is likely attributed to the increased vascular permeability of peritoneal metastatic foci and the EPR effect characteristic of peritoneal metastases [71]. The integration of fluorescence diagnosis with artificial intelligence algorithms during laparoscopy demonstrated a substantial improvement in detecting peritoneal metastases in an experimental model, supporting the promise of a fluorescence guided approach for the diagnosis and treatment of PC [72]. Confocal fluorescence microscopy data of biopsy samples obtained in the present study verify the specificity of Ce6 accumulation in metastatic foci at the cellular level.

The feasibility of utilizing a single platform across such diverse clinical scenarios confirms the modularity and versatility of the VENERA-red-660 system design, distinguishing it favorably from most previously reported systems. Employing a single 660 nm laser simultaneously as both a therapeutic and diagnostic source simplifies the operational setup and reduces the financial burden compared to platforms requiring separate sources for fluorescence excitation and PDT.

## 5. Limitations

This study has several limitations that should be considered when interpreting the results. The pilot nature of the work and the small sample size preclude drawing statistically robust conclusions regarding treatment efficacy, necessitating prospective studies with an expanded patient cohort. Long-term clinical treatment outcomes (complete response rate, time to progression, and survival) were not evaluated in the present work and require a separate prospective analysis with an extended follow up period.

## 6. Conclusions

We developed and clinically evaluated VENERA-red-660, a new dual-channel endoscopic video-fluorescence system that images the anatomical scene and Ce6 photosensitizer distribution simultaneously, at λ_ex_ = 660 nm, without interrupting PDT irradiation. This distinguishes it from existing endoscopic fluorescence platforms, which require pausing therapy to switch between white-light and fluorescence modes.

In a pilot cohort of 18 patients spanning three clinically distinct scenarios: external access (BCC, VIN III) and laparoscopic access (PC recurrent HGSOC), combined with PIPAC), the system maintained a linear fluorescence response down to the lowest tested concentration (0.1 mg/L; R^2^ = 0.998) and tracked a decrease in Ce6 fluorescence consistent with photobleaching (FCI reduction of 57%, 66%, and 60% in the BCC, VIN III, and PC groups, respectively). These results indicate that VENERA-red-660 can support real-time PDT dosimetry across a range of surgical access types within a single protocol, rather than requiring separate instrumentation for open, endoscopic, and laparoscopic procedures.

These pilot findings support VENERA-red-660 as a practical tool for intraoperative fluorescence diagnostics and treatment monitoring during PDT using chlorin-based PSs. Validation in larger, prospective cohorts with longer follow-up is needed to establish its impact on treatment outcomes.

## Figures and Tables

**Table 1:** Inclusion and exclusion criteria for each patient cohort.

Category	BCC	VIN III	PC
Inclusion criteria	Written informed consent to participate; age over 18 years; histologically confirmed BCC	Women aged 18 years and older; HPV-associated vulvar intraepithelial neoplasia (HSIL/VIN III) or HPV-associated malignant neoplasm of the vulva (TisN0M0, preinvasive vulvar carcinoma); written informed consent to participate	Written informed consent to participate; age over 18 years; female sex; morphologically verified recurrent ovarian cancer (stage III); ECOG performance status 0–2
Exclusion criteria	Hypersensitivity to the photosensitizer; severe renal or hepatic insufficiency; decompensated cardiovascular disease (chronic heart failure, arterial hypertension); pregnancy; lactation; patient refusal of treatment	Pregnancy and lactation; psychiatric disorders; hereditary or acquired porphyria; increased cutaneous photosensitivity; presence of severe concomitant somatic disease; hepatic or renal insufficiency; invasive carcinoma ≥ stage IA1; occurrence of significant events precluding continued study participation; inability to continue the study intervention for non-medical reasons	Withdrawal of patient consent to further participation; pregnancy; acute coronary syndrome, acute circulatory disturbance, or other condition requiring resuscitation; age under 18 years; presence of psychiatric disorders; acute venous thrombosis; ECOG performance status 3–4

Abbreviations: BCC—basal cell carcinoma; VIN III—vulvar intraepithelial neoplasia grade III; PC—peritoneal carcinomatosis; ECOG—Eastern Cooperative Oncology Group (performance status); HPV—human papillomavirus; HSIL—high-grade squamous intraepithelial lesion.

**Table 2:** Clinically approved chlorin-based photosensitizers and their absorption and fluorescence spectral characteristics.

Photosensitizer	Chemical Name	Medium	Soret Band Absorption	Red Absorption	Fluorescence Peak	Reference
Photolon^®^/Fotoran E6^®^	Trisodium salt of Ce6	Human blood plasma	~408 nm	~664 nm	~672 nm	Our data
Fotoditazin^®^	Dimeglumine sodium salt of Ce6	OctOH	~403 nm	~665 nm	~671 nm	[16]
Radachlorin^®^/Bremachlorin^®^	Mixture of sodium salts of Ce6, chlorin p6, and purpurin 5	HSA	~405 nm	~662 nm	~670 nm	[35]
Foscan^®^ (Temoporfin)	mTHPC	RPMI 1640 cell culture with 10% FBS	~424 nm	~650 nm	~650 nm	[36]
Laserphyrin^®^ (Talaporfin)	NPe6	PBS	~406 nm	~653 nm	~660 nm	[37]

Abbreviations: Ce6—chlorin e6; OctOH—1-octanol; FBS—fetal bovine serum; HSA—human serum albumin; mTHPC—5,10,15,20-Tetra(m-hydroxyphenyl) chlorin; NPe6—mono-L-aspartyl chlorin e6; PBS—phosphate-buffered saline.

## Data Availability

The data presented in this study are available on request from the corresponding author.

## Abbreviations

The following abbreviations are used in this manuscript:

PDT	Photodynamic therapy
PS	Photosensitizer
Ce6	Chlorin e6
BCC	Basal cell carcinoma
VIN III	Vulvar intraepithelial neoplasia grade III
PC	peritoneal carcinomatosis
HGSOC	High-grade serous ovarian carcinoma
FCI	Fluorescence contrast index
PIPAC	Pressurized intraperitoneal aerosol chemotherapy
HPV	Human papillomavirus
5-ALA	5-aminolevulinic acid
HAL	Hexaminolevulinate
MAL	Methylaminolevulinate
PpIX	Protoporphyrin IX
IOPDT	Intraoperative PDT
HIPEC	Hyperthermic intraperitoneal chemotherapy
PCI	Peritoneal cancer index
